# Unmet clinical laboratory need in patients hospitalized for acute poisoning from long-acting anticoagulant rodenticides

**DOI:** 10.1080/24734306.2021.1925444

**Published:** 2021-06-04

**Authors:** Richard B. van Breemen, John W. Hafner, Daniel G. Nosal, Douglas L. Feinstein, Israel Rubinstein

**Affiliations:** a Linus Pauling Institute, Oregon State University, Corvallis, OR, USA; b Department of Emergency Medicine, University of Illinois, Peoria, IL, USA; c Department of Anesthesiology, University of Illinois, Chicago, IL, USA; d Jesse Brown VA Medical Center, Chicago, IL, USA; e Department of Medicine, University of Illinois, Chicago, IL, USA

**Keywords:** bleeding, INR, blood transfusion, clotting factor concentrate, vitamin K1, synthetic cannabinoids

## Abstract

The importance of real-time, quantitative toxicology data available for physicians treating poisoned patients was illustrated during the 2018 outbreak in Illinois of severe coagulopathy caused by inhaling illicit synthetic cannabinoids products contaminated with commercially-available brodifacoum, difenacoum, and bromadiolone, three potent, long-acting anticoagulant rodenticides (LAARs). Identification and quantification of these life-threatening toxins in blood samples of hospitalized patients required toxicology testing with liquid chromatography-tandem mass spectrometry (LC-MS/MS) that was not available in clinical laboratories of hospitals at the time of the outbreak. This highly-sensitive, quantitative assay can provide critical information to guide patient care during and after hospitalization, including identification of offending LAARs, estimates of the ingested dose, and dosage and discontinuation of oral vitamin K_1_ therapy after hospital discharge once plasma LAARs concentrations decreased to a safe level (<10 ng/mL). Accordingly, we propose an action plan to enable treating physicians to quantify plasma concentrations of several LAARs simultaneously in poisoned patients. It involves rapid (<15 min), sensitive, and validated LC-MS/MS methods developed, tested and validated in our laboratory. This will allow treating physicians to request quantitative plasma LAARs testing, report test results in the patient’s hospital discharge summary, and recommend regular monitoring of plasma LAARs concentrations in the outpatient setting.

## The clinical challenge

The importance of real-time, quantitative toxicology data available for physicians treating poisoned patients was illustrated during the 2018 outbreak in Illinois of severe coagulopathy provoked by inhaled illicit synthetic cannabinoids products contaminated with commercially-available brodifacoum, difenacoum, and bromadiolone, three potent, long-acting anticoagulant rodenticides (LAARs) [1-3].

Identification and quantification of these life-threatening toxins in blood samples of patients hospitalized for acute poisoning required sophisticated toxicology testing using liquid chromatography-tandem mass spectrometry (LC-MS/MS) that was not available in clinical laboratories of hospitals at the time of the outbreak [4-7]. Accordingly, we propose that this highly-sensitive quantitative assay for plasma LAARs should be available to physicians treating patients with acute LAARs poisoning because it can provide critical information to guide patient care during and after hospitalization, including identification of the offending LAARs, estimates of the ingested dose, and assistance in determining dose and treatment duration of oral vitamin K_1_ after hospital discharge [8]. To that end, premature discontinuation of oral vitamin K_1_ during follow-up when coagulation studies are within the normal range may result in recurrence of coagulopathy, bleeding, and death [9,10]. Regular monitoring of plasma LAARs concentrations until a safe level (<10 ng/mL) is reached could then mitigate this ominous clinical scenario [4-6].

### Unmet clinical laboratory need

Unfortunately, anticoagulant poison panels that quantify LAARs in human plasma, including brodifacoum, difenacoum, and bromadiolone, are not available in hospitals at the present time [11]. This, in turn, prevents physicians from ordering the assay in patients with severe coagulopathy-associated bleeding of unknown etiology. To that end, two Clinical Laboratory Improvement Amendments (CLIA)-certified laboratories, Wisconsin State Laboratory of Hygiene (Madison, WI, USA), a Laboratory Response Network-C Level 1 facility that tests samples in the event of a large-scale chemical emergency, and NMS Labs (Horsham, PA, USA), a commercial laboratory, have now developed a quantitative test for plasma brodifacoum determination with a relatively rapid turnaround time [12,13], and both reference laboratories now offer such testing nationally. Accordingly, physicians can diagnose and appropriately treat hospitalized patients with acute brodifacoum poisoning and recommend regular monitoring of plasma brodifacoum concentrations in the outpatient settings until a concentration considered safe (<10 ng/mL) is recorded [4-6].

However, at the present time, neither of the two reference laboratories provides testing for plasma concentrations of other potent LAARs such as difenacoum and bromadiolone. This deficiency represents a life-threatening, unmet medical need because difenacoum, bromadiolone and brodifacoum were detected in blood during the recent outbreak in Illinois and other States [1-3]. Hence, quantitative determination of difenacoum and bromadiolone, in addition to brodifacoum, in blood samples from patients hospitalized for suspected acute LAARs poisoning should be urgently made available to physicians.

### Proposed action plan

We propose an action plan that will enable physicians to quantify simultaneously plasma concentrations of brodifacoum, difenacoum, bromadiolone, and other LAARs in poisoned patients. The approach involves rapid, sensitive and validated LC-MS/MS methods that were developed and validated in our laboratory as outlined below [14-16].

Analytical method validation involves evaluating and controlling variables such as lower and upper limits of quantitation, analyte stability, extraction recovery, matrix interference, accuracy, and precision according to guidelines established by organizations such as the U.S. Food and Drug Administration (FDA) [11]. To date, all the validated LC-MS/MS methods developed in our laboratory have lower limits of quantitation (LOOQ) for LAARs of <3 ng/mL. Accordingly, they are suitable for the purpose of determining if blood LAARs concentrations exceed 10 ng/mL, above which vitamin K_1_ supplementation is required to prevent coagulopathy [14-16]. Although gas chromatograph MS (GC-MS) methods exist to quantify some LAARs in blood samples, to date there are no GC-MS methods reported for quantitative and simultaneous analysis of multiple LAARs in blood samples.

Our new LC-MS/MS methods for quantitative analysis of LAARs in blood samples require as little as 100 μL of whole blood or plasma and make use of a highly efficient extraction step [14]. Some methods have used solid phase extraction for sample preparation, but the highest extraction recoveries of multiple LAARs from blood (>80% recovery) have utilized simple protein precipitation with methanol and acetonitrile [14,15]. Although dried blood-spot analyses would provide greater convenience with respect to sample handling, this alternative to liquid-liquid extraction of blood or plasma is not yet available for the quantitative analysis of LAARs.

**Figure 1.**
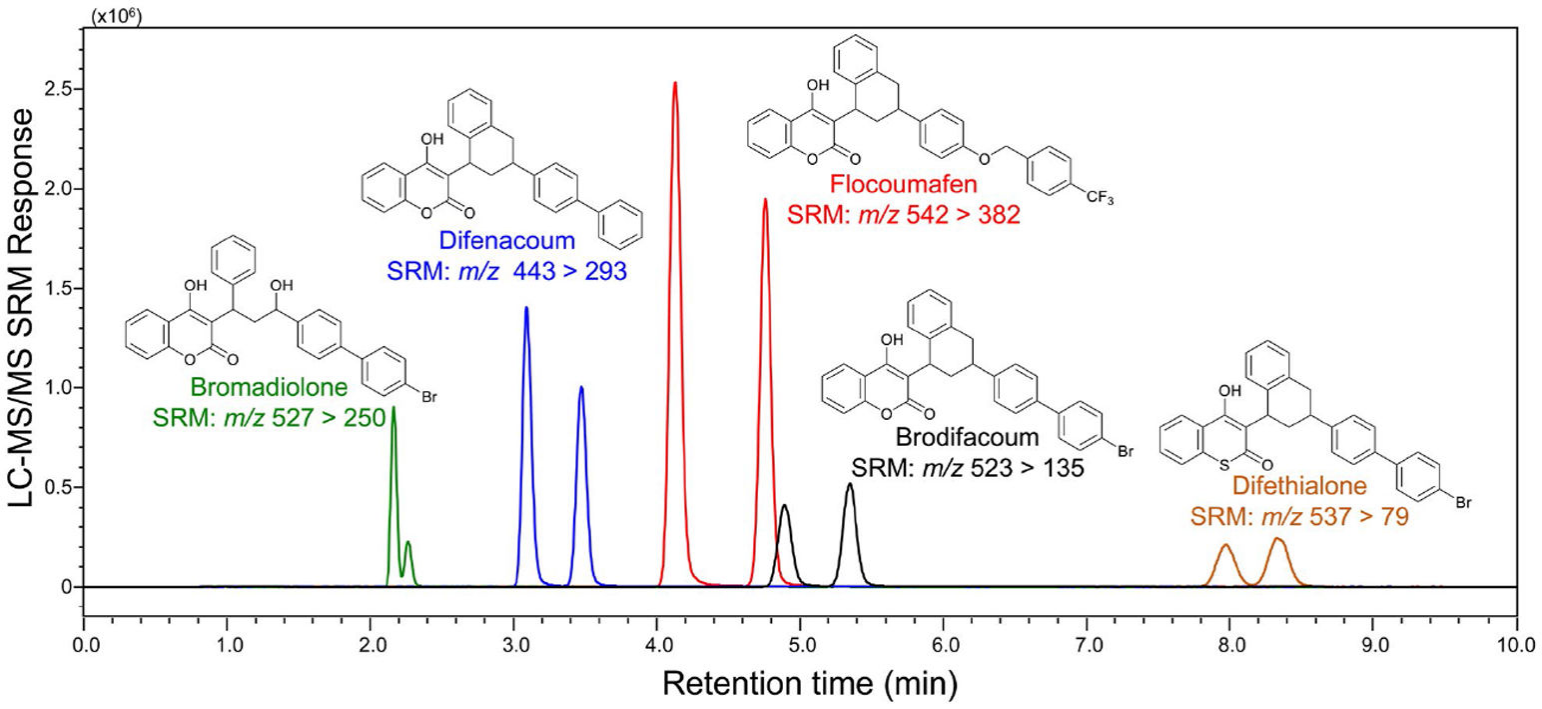
After treatment of whole blood or plasma with organic solvent to precipitate proteins and then centrifugation, the supernatant is analyzed using reversed phase ultrahigh-pressure liquid chromatography-tandem mass-spectrometry (UHPLC-MS/MS). in this example, the long-acting anticoagulant rodenticides (LAARs) brodifacoum, bromadiolone, difenacoum, difethialone, and flocoumafen were extracted from 100 μL human plasma, separated using reversed phase UHPLC and measured in a single analysis using negative ion electrospray tandem mass spectrometry with selected-reaction monitoring (SRM). Note that each racemic LAAR was detected as a pair of cis/trans diastereomers due to the presence of two chiral centers.

Most validated LC-MS/MS methods for LAARs use C_18_ reversed phase chromatographic separation with either standard HPLC columns or ultrahigh-pressure liquid chromatography [UHPLC] (Figure 1) [14-16]. The UHPLC methods provide both higher chromatographic resolution and faster analysis (<10 min). When using methanol in the mobile phase, single chromatographic peaks are detected for each LAAR [7] even though most LAARs including brodifacoum, difenacoum and bromadiolone contain two chiral carbons and are marketed as racemic mixtures. In contrast, the use of acetonitrile instead of methanol during HPLC or UHPLC enables separation of LAARs into pairs of diastereomers [14,15]. Since, like warfarin, different LAAR diastereomers have distinct anticoagulant properties and half-lives, knowledge of specific diastereomer concentrations can be of clinical relevance [14,15]. To enable even greater understanding of the blood levels of individual LAAR stereoisomers, an LC-MS/MS method was recently reported that uses chiral instead of reversed phase chromatography to separate all four stereoisomers of brodifacoum, difenacoum, and bromadiolone for quantitative analysis during a single analysis in less than 12 min [Figure1] [16].

All current mass spectrometry-based methods for the quantitative analysis of LAARs in human blood use electrospray for ionization, and negative ion electrospray is preferred to positive ion mode due to lower background noise and lower limits of quantitation (Figure 1) [14-16]. Atmospheric pressure chemical ionization, an alternative LC-MS ionization technique, has been evaluated for the analysis of LAARs but was found to provide lower sensitivity than electrospray, especially for brodifacoum [7]. Although high resolution mass spectrometers such as Orbitraps have been used for quantitative analysis of LAARs, preferred methods achieve higher sensitivity using selected reaction monitoring on less costly triple quadrupole mass spectrometers [14-16].

### Clinical implications

During the 2018 outbreak in Illinois, treating physicians and the Illinois Poison Center were at a loss for exactly how long to monitor patients exposed to LAARs. It was unclear if the LAARs themselves underwent any substantial pyrolysis during smoking of the synthetic cannabinoids, or what the effective exposure dose was. A lack of easily obtainable quantitative LAARs concentrations in the blood of victims may have resulted in more outpatient coagulation testing and importantly, more costly, and lengthy oral vitamin K_1_ supplementation than necessary [1-3,6]. To that end, the qualitative LC-MS/MS LAARs assay offered by NMS Labs [13] to treating physicians during the outbreak in Illinois can detect only brodifacoum in blood samples and is inefficient for monitoring poisoned patients over time as several tests would have been required until blood concentrations dropped to <10 ng/mL, the detection level of the assay [4-6,12,13]. By contrast, our rapid (<15 min), highly-sensitive, quantitative, and validated LC-MS/MS assay can provide critical information to guide patient care during and after hospitalization, including identification of several offending LAARs, estimates of the ingested dose, and dosage and discontinuation of oral vitamin K_1_ therapy after hospital discharge once plasma LAARs concentrations decreased to a safe level (<10 ng/mL) [6,9,10,14-16]. Treating physicians will request quantitative plasma LAARs testing, report test results in the patient’s hospital discharge summary, and recommend regular monitoring of plasma LAARs concentrations in the outpatient setting.

### Take home message

In summary, we propose that physicians treating patients with acute poisoning from LAARs should request quantitative testing for plasma brodifacoum, difenacoum, and bromadiolone concentrations (Figure 1), report these test results in hospital discharge instructions given to patients, and recommend regular monitoring of their plasma concentrations in the outpatient setting.
